# Renal Function and Risk Factors of Moderate to Severe Chronic Kidney Disease in Golestan Province, Northeast of Iran

**DOI:** 10.1371/journal.pone.0014216

**Published:** 2010-12-03

**Authors:** Iraj Najafi, Fatemeh Attari, Farhad Islami, Ramin Shakeri, Fatemeh Malekzadeh, Rasool Salahi, Mina Yapan Gharavi, Mostafa Hosseini, Behrooz Broumand, Ali Nobakht Haghighi, Bagher Larijani, Reza Malekzadeh

**Affiliations:** 1 Nephrology Research Center, Shariati Hospital, Tehran University of Medical Sciences, Tehran, Iran; 2 Digestive Disease Research Center, Shariati Hospital, Tehran University of Medical Sciences, Tehran, Iran; 3 International Agency for Research on Cancer, Lyon, France; 4 Golestan Research Center of Gastroenterology and Hepatology, Golestan University of Medical Sciences, Gorgan, Iran; 5 School of Public Health, Department of Epidemiology and Biostatistics, Tehran University of Medical Sciences, Tehran, Iran; 6 Clinical Science Study Group Iran, IR of Iran Academy of Medical Sciences, Tehran, Iran; 7 Endocrinology and Metabolism Research Center, Shariati Hospital, Tehran University of Medical Sciences, Tehran, Iran; Kenya Medical Research Institute, Kenya

## Abstract

**Introduction:**

The incidence of end-stage renal disease is increasing worldwide. Earlier studies reported high prevalence rates of obesity and hypertension, two major risk factors of chronic kidney disease (CKD), in Golestan Province, Iran. We aimed to investigate prevalence of moderate to severe CKD and its risk factors in the region.

**Methods:**

Questionnaire data and blood samples were collected from 3591 participants (≥18 years old) from the general population. Based on serum creatinine levels, glomerular filtration rate (GFR) was estimated.

**Results:**

High body mass index (BMI) was common: 35.0% of participants were overweight (BMI 25–29.9) and 24.5% were obese (BMI ≥30). Prevalence of CKD stages 3 to 5 (CKD**–**S3-5), i.e., GFR <60 mL/min/1.73 m^2^, was 4.6%. The odds ratio (OR) and 95% confidence interval (95% CI) for the risk of CKD**–**S3-5 associated with every year increase in age was 1.13 (1.11–1.15). Men were at lower risk of CKD**–**S3-5 than women (OR = 0.28; 95% CI 0.18–0.45). Obesity (OR = 1.78; 95% CI 1.04–3.05) and self-reported diabetes (OR = 1.70; 95% CI 1.00–2.86), hypertension (OR = 3.16; 95% CI 2.02–4.95), ischemic heart disease (OR = 2.73; 95% CI 1.55–4.81), and myocardial infarction (OR = 2.69; 95% CI 1.14–6.32) were associated with increased risk of CKD**–**S3-5 in the models adjusted for age and sex. The association persisted for self-reported hypertension even after adjustments for BMI and history of diabetes (OR = 2.85; 95% CI 1.77–4.59).

**Conclusion:**

A considerable proportion of inhabitants in Golestan have CKD**–**S3-5. Screening of individuals with major risk factors of CKD, in order to early detection and treatment of impaired renal function, may be plausible. Further studies on optimal risk prediction of future end-stage renal disease and effectiveness of any screening program are warranted.

## Introduction

Kidney disease contributes to a significant morbidity and mortality in societies [1]. The incidence of chronic kidney disease (CKD) and end-stage renal disease (ESRD) is increasing [2], . According to the National Health and Nutrition Examination Surveys (NHANES), while prevalence of CKD (excluding ESRD) in the United States was 10.0% in 1988–1994, the prevalence increased to 13.1% in 1999–2004 [4].

In addition to morbidity and common psychosocial outcomes of the disease, including depression and unemployment, ERSD patients need dialysis or transplantation facilities, which may place a big financial burden on the society. In the past decade, 1.1 trillion dollars have been spent for dialysis worldwide [5]. Early diagnosis and treatment of mild to moderate CKD may prevent or delay progression of the disease to severer stages [2],[3],[6],[7],[8]. Therefore, diagnosis and treatment of CKD in early stages can be an important public health issue, particularly in the developing countries with high rates of CKD. Nevertheless, little data on epidemiology of renal function and abnormalities are available from many of those countries [9].

Among very few population-based studies on kidney disease in Iran, a recent large study reported that prevalence of CKD among individuals >14-years old in different parts of the country was 6% to 17% [10]. In Golestan Province, northeast of Iran, very high rates of overweight/obesity and hypertension have recently been reported among participants in a large-scale cohort study [11]. In general, CKD is more common in older ages, obese individuals, and certain medical conditions, including diabetes and hypertension [12], [13], [14]. Therefore, Golestan inhabitants might be highly predisposed to CKD. We conducted a population-based cross-sectional study in Golestan and collected a wide range of data to evaluate renal function, based on estimated glomerular filtration rate (GFR), in the general population and to investigate risk factors of moderate to severe CKD in the region.

## Methods

A pilot trial of the effects of fixed-dose combination therapy (polypill) on cardiovascular risk factors was conducted recently in Kalaleh City, Golestan Province [15]. For that study, all men aged 50 to 79 and women aged 55 to 79 years residing in Kalaleh City were invited to attend initial assessments of their eligibility for the trial. The number of inhabitant aged 18 years or older in the study field was 18,218, of which 2427 were in the above specified age ranges. Excluding those whom we could not contact or were unable to attend in health center (305 subjects), Questionnaire data and blood samples were collected from all of those who came to the study center (Kalaleh Health Center) and agreed to provide the material (n = 1488; participation rate 70%). Eligible individuals were invited to participate in the pilot trial later. Data and samples from all of the above individuals were used for the current study, without considering whether or not they were included in the pilot trial. In order to increase the sample size and study power, the study participants were contacted by phone and all individuals aged 18 years or older in their household were invited to participate in the study. From those who agreed, the same data and samples as above were obtained. With a participation rate of over 95% in this group, a total number of 3613 participants were enrolled in the study from April 2007 to January 2009. A written informed consent was obtained from all participants. The study protocol and the informed consent used for this study were approved by the ethical review committee of Digestive Disease Research Center of Tehran University of Medical Sciences.

Demographic data and history of medical conditions, including diabetes mellitus, hypertension, ischemic heart disease, and myocardial infarction, were collected in face to face interviews using a structured questionnaire. Weight and height were measured by interviewers. Systolic and diastolic blood pressures were obtained twice for both arms during the interviews; only measurements for right arm were considered for further analyses. Interviews and physical examinations were conducted by trained physicians.

Blood samples were drawn after at least 10-hour overnight fast and were centrifuged within 1 hour of collection. Serum samples were separated and were kept in −70°C freezers in the study center for a maximum period of three months. The samples were transported in dry ice to Endocrinology Research Center of Tehran University of Medical Sciences, Shariati Hospital, in Tehran, where serum creatinine and fasting blood sugar were measured. The duration of transportation was less than 10 hours and none of the samples were found unfrozen at destination, where the samples were immediately put in −70°C freezers. The laboratory analyses were done within a few days after arrival with endpoint enzymatic photometric method using Hitachi 902 auto-analyzer. An expert nephrologist attended the Kalaleh Health Center in a monthly basis and visited the participants who had any abnormal test. When results of laboratory tests were abnormal, blood sample collection and tests were repeated approximately three months later. In this article, we considered a test as abnormal when it was confirmed in a repeated test. Individuals with abnormal tests were referred to their family physicians. All family physicians and internists in the study field had been invited to attend a lecture by the principal investigator of the study in the beginning, in which they were told about the study goals and protocol.

Body mass index (BMI) was categorized according to the World Health Organization guidelines: <18.5 (underweight), 18.5–24.9 (normal), 25–29.9 (overweight), and ≥30 (obese) kg/m^2^. Participants were classifed as diabetics when they had either self-reported diabetes (diagnosed by a physician and/or receiving medications for diabetes) or fasting blood sugar ≥110 mg/dL. Hypertension was defined as any of these conditions: (1) self-reported hypertension (diagnosed by a physician or receiving medications for hypertension); (2) average systolic blood pressure ≥140 mmHg; (3) average diastolic blood pressure ≥90 mmHg.

To estimate GFR, we used Modification of Diet in Renal Disease (MDRD) study equation: GFR [mL/min/1.73 m^2^]  = 186× (serum creatinine [mg/dL])^-1.15^× (age [years])^-0.203^ ×0.742 (if female) ×1.212 (if African-American) [16]. We also estimated GFR using Cockroft-Goult equation [17]: Creatinine Clearance [ml/min]  =  ((140 – age [years])/serum creatinine [mg/dL]) x (weight [kg]/72) ×0.85 (if female); the values were standardized for body surface area calculated using Du Bios Method [18]: body surface area [m^2^]  = 0.20247×0.725 height [m] ×0.425 weight [kg]. According to one of the most widely adopted classifications, proposed by the Kidney Disease Outcomes Quality Initiative, 5 stages have been defined for CKD [3]. In stage 1 and stage 2, markers of kidney damage, including increased urinary excretion of albumin and protein, are present and glomerular filtration rates (GFR) are ≥90 and 60–89 mL/min/1.73 m^2^, respectively. Higher stages are defined only by GFR, which is 30–59, 15–29, and <15 mL/min/1.73 m^2^ in stages 3, 4, and 5, respectively. In accord with above categories, we classified the estimated GFR into 5 groups: >90, 60–90, 30–59, 15–29, and <15 mL/min/1.73 m^2^.

### Statistical analysis

We investigated the association between stages 3 to 5 of CKD (CKD**–**S3-5) and several factors, including demographic characteristics, BMI, and history of diabetes and hypertension. For this, individuals with GFR <60 mL/min/1.73 m^2^ were combined and compared with those having GFR >90 mL/min/1.73 m^2^. Participants with estimated GFR of 60–90 mL/min/1.73 m^2^ were not included in these analyses. We calculated crude and adjusted odds ratios (OR) and 95% confidence intervals (CI) for the associations in logistic regression models. For diabetes and hypertension, we investigated the association separately for those with self-reported disease, those who had abnormal findings in baseline examinations, and both categories combined. All of the statistical analyses were done using STATA version 11.

## Results

Of 3613 enrolled participants, 22 (0.6%) were excluded because their laboratory analyses were not completed. Consequently, 3591 individuals were included for further analyses. The number and mean (standard deviation) age of participants by demographic characteristics, BMI, and history of medical conditions is shown in Table 1. Mean (SD) age was 43.8 (15.8) years. Sixty one percent of participants were women. Many participants had high BMI: 1309 (36.5%) were overweight (BMI  = 25–29.9 kg/m^2^) and 881 (24.5%) were obese (BMI ≥30 kg/m^2^). Diabetes, hypertension, ischemic heart disease, and myocardial infarction were reported by 14.3%, 20.5%, 9.1%, and 3.8% of participants, respectively. Prevalence of diabetes and hypertension, either self-reported or identified in baseline examinations, was 21.6% and 39.9%, respectively.

**Table 1:** Distribution of demographic variables, BMI, and history of several medical conditions among study participants.

	Number (%)	Mean age (SD), years
**All participants**	3591 (100)	43.8 (15.8)
**Women**	2192 (61.0)	41.2 (15.1)
**Men**	1399 (39.0)	47.9 (15.9)
**Age (years)**		
<30	847 (23.6)	23.1 (3.5)
30–39	656 (18.3)	34.5 (2.8)
40–49	634 (17.7)	44.4 (3.0)
50–59	806 (22.4)	54.2 (2.9)
60–69	433 (12.0)	63.8 (3.0)
≥70	215 (6.0)	72.5 (2.4)
**Ethnicity**		
Sistani	1513 (42.1)	42.4 (15.4)
Turkmen	762 (21.2)	46.6 (15.4)
Fars	864 (24.1)	43.7 (15.9)
Other	452 (12.6)	44.2 (16.6)
**Marital Status**		
Married	2868 (79.9)	46.1 (14.1)
Single	483 (13.4)	23.3 (6.4)
Widow/divorced	240 (6.7)	58.2 (12.4)
**Education**		
No school	1205 (33.5)	57.7 (10.0)
Primary school	732 (20.4)	47.7 (11.8)
Middle school	394 (11.0)	36.9 (11.0)
High school	891 (24.8)	29.3 (10.1)
University	369 (10.3)	33.3 (10.8)
**BMI**		
<18.5	145 (4.0)	34.1 (17.9)
18.5–24.9	1309 (36.5)	40.5 (17.7)
25–29.9	1256 (35.0)	46.5 (14.1)
≥30	881 (24.5)	46.5 (12.8)
**Diabetes ^c^**		
Self-reported	513 (14.3)	45.1 (15.3)
FBS ≥110 mg/dL	497 (13.8)	54.8 (11.0)
Any criteria	777 (21.6)	48.4 (14.8)
**Hypertension ^c^**		
Self-reported	736 (20.5)	48.3 (15.7)
Sys ≥140 or dias ≥90 mmHg	1073 (29.9)	52.4 (13.2)
Any criteria	1432 (39.9)	49.4 (14.5)
**Ischemic heart disease**	327 (9.1)	47.6 (16.7)
**Myocardial infarction**	138 (3.8)	43.1 (15.2)

Abbreviations: BMI, body mass index (kg/m^2^); SD, standard deviation.

Distribution of MDRD-estimated GFR categories by sex and age is presented in Table 2. GFR remarkably reduced with age. For example, the proportion of individuals with GFR 30–59 mL/min/1.73 m^2^ increased from 0.5% among participants below 30 years of age to 20% in those with 70 years of age or more, while the proportion of individuals with normal GFR in those age categories were 76% and 10%, respectively. The distribution for GFRs calculated using Cockroft-Goult is shown in Table S1. The GFRs based on Cockroft-Goult method were generally lower than those based on MDRD method. As Cockroft-Goult method may underestimate the GFR [19], we used only MDRD-based GFRs for further analyses.

**Table 2:** MDRD-based Glomerular filtration rate by sex and age.

	Mean age (SD), years	Total no.	GFR ≥90	GFR: 60–89	GFR: 30–59	GFR: 15–29	GFR <15
All participants	43.8 (15.8)	3591	1240 (34.5)	2186 (60.9)	154 (4.3)	6 (0.2)	5 (0.1)
Women	41.2 (15.1)	2192	721 (32.9)	1367 (62.4)	99 (4.5)	4 (0.2)	1 (0.1)
Men	48.0 (15.9)	1399	519 (37.1)	819 (58.5)	55 (3.9)	2 (0.1)	4 (0.3)
Age (years)							
<30	23.1 (3.5)	847	647 (76.4)	194 (22.9)	4 (0.5)	1 (0.1)	1 (0.1)
30–39	34.5 (2.8)	656	186 (28.3)	465 (70.9)	5 (0.8)	0 (0.0)	0 (0.0)
40–49	44.4 (3.0)	634	117 (18.4)	502 (79.2)	14 (2.2)	1 (0.2)	0 (0.0)
50–59	54.2 (2.9)	806	184 (22.8)	593 (73.6)	26 (3.2)	2 (0.3)	1 (0.1)
60–69	63.8 (3.0)	433	84 (19.4)	284 (65.6)	62 (14.3)	2 (0.5)	1 (0.2)
≥70	72.5 (2.4)	215	22 (10.2)	148 (68.8)	43 (20.0)	0 (0.0)	2 (0.9)

Abbreviations: BMI, body mass index (kg/m^2^); GFR, glomerular filtration rate (ml/min/1.73 m^2^); MDRD, Modification of Diet in Renal Disease; SD, standard deviation.

Values are numbers (percentages) of participants unless stated otherwise.

Table 3 shows the association of CKD**–**S3-5 (MDRD-based GFR <60 mL/min/1.73 m^2^) with demographic factors, BMI, and history of diabetes, hypertension, ischemic heart disease, and myocardial infarction. One hundred sixty seven participants (4.6% of all participants) had CKD**–**S3-5. Except for ethnicity, all other variables in Table 2 showed an association with CKD**–**S3-5 in crude analyses. However, after adjustments for age and sex, the associations for marital status and education level were disappeared. The OR (95% CI) for every year increase in age was 1.13 (1.11–1.15) in adjusted models. Men were at lower risk than women after adjustment for age (OR = 0.24; 95% CI 0.16–0.38) or after extension of the adjustment to BMI and self-reported diabetes and hypertension (OR = 0.28; 95% CI 0.18–0.45).

**Table 3:** Association of CKD stages 3 to 5 (GFR <60) with demographic characteristics, BMI, and history of selected diseases in logistic regression models.

Variable	GFR ≥90 (%)n = 1240	CKD stages 3–5 (%)n = 165	Crude OR(95% CI)	Adjusted OR(95% CI) a	Adjusted OR(95% CI) b
**Age**, year (continuous)	-	-	1.12 (1.10–1.14)	1.13 (1.11–1.15)	1.13 (1.11–1.15)
**Sex**					
Women	721 (58.2)	104 (63.0)	Reference	Reference	Reference
Men	519 (41.8)	61 (37.0)	0.81 (0.58–1.14)	0.24 (0.16–0.38)	0.28 (0.18–0.45)
**Ethnicity**					
Sistani	516 (41.6)	58 (35.2)	Reference	Reference	Reference
Turkmen	254 (20.5)	40 (24.2)	1.40 (0.91–2.15)	0.96 (0.56–1.64)	0.83 (0.47–1.45)
Fars	301 (24.3)	42 (25.5)	1.24 (0.81–1.89)	0.99 (0.58–1.68)	0.78 (0.44–1.38)
Other	169 (13.6)	25 (15.1)	1.32 (0.80–2.17)	1.01 (0.54–1.90)	0.72 (0.37–1.40)
**Marital Status**					
Married	845 (68.2)	130 (78.8)	Reference	Reference	Reference
Single	354 (28.5)	2 (1.2)	0.04 (0.01–0.15)	0.42 (0.08–2.12)	0.52 (0.10–2.65)
Widow/divorced	41 (3.3)	33 (20.0)	5.23 (3.19–8.58)	0.80 (0.42–1.53)	0.75 (0.39–1.47)
**Education**					
No school	238 (13.2)	114 (69.1)	Reference	Reference	Reference
Primary school	171 (13.8)	29 (17.6)	0.35 (0.23–0.57)	1.37 (0.78–2.41)	1.22 (0.68–2.18)
Middle school	150 (12.1)	8 (4.8)	0.11 (0.05–0.23)	1.79 (0.70–2.56)	1.58 (0.61–4.05)
High school	514 (41.4)	10 (6.1)	0.04 (0.02–0.08)	1.56 (0.61–3.94)	1.48 (0.57–3.83)
University	167 (13.5)	4 (2.4)	0.05 (0.02–0.14)	1.63 (0.49–5.40)	1.43 (0.43–4.80)
**BMI**					
<18.5	100 (48.0)	2 (33.3)	0.21 (0.05–0.90)	0.44 (0.09–2.13)	0.46 (0.08–2.20)
18.5–24.9	595 (8.0)	55 (1.2)	Reference	Reference	Reference
25–29.9	338 (27.3)	58 (35.2)	1.86 (1.25–2.75)	1.55 (0.93–2.57)	1.57 (0.78–2.19)
≥30	207 (19.7)	50 (30.3)	2.61 (1.73–3.95)	1.78 (1.04–3.05)	1.35 (0.77–2.36)
*P* for trend			<0.001	0.01	0.15
**Diabetes** c					
Self-reported	161 (13.0)	32 (19.4)	1.61 (1.06–2.45)	1.70 (1.00–2.86)	1.13 (0.65–1.98)
FBS ≥110 mg/dL	116 (9.4)	43 (26.1)	3.42 (2.30–5.08)	1.24 (0.76–2.00)	0.94 (0.56–1.57)
Any criteria	225 (18.2)	54 (32.7)	2.19 (1.54–3.13)	1.47 (0.94–2.30)	1.07 (0.66–1.72)
**Hypertension** c					
Self-reported	182 (14.7)	71 (43.0)	4.39 (3.11–6.21)	3.16 (2.02–4.95)	2.85 (1.77–4.59)
Sys ≥140 or dias ≥90 mmHg	243 (19.6)	83 (50.3)	4.15 (2.97–5.81)	1.43 (0.93–2.18)	1.26 (0.81–1.94)
Any criteria	356 (28.7)	100 (60.6)	3.80 (2.71–5.33)	2.64 (1.50–2.78)	1.61 (1.04–2.50)
**IHD** c	87 (7.0)	39 (23.6)	4.10 (2.70–6.24)	2.73 (1.55–4.81)	1.73 (0.93–3.21)
**MI** c	47 (3.8)	13 (7.9)	2.17 (1.15–4.10)	2.69 (1.14–6.32)	1.55 (0.61–3.92)

Abbreviations: BMI, body mass index (kg/m^2^); CI, confidence interval; CKD, chronic kidney disease; dias, diastolic blood pressure; FBS, fasting blood sugar; GFR, glomerular filtration rate (ml/min/1.73 m^2^); IHD, ischemic heart disease; MI, myocardial infarction; OR, odds ratio; sys, systolic blood pressure.

a Adjusted for sex and age (categorical variable). Sex variable was only adjusted for age and vice versa.

b Adjusted for sex, age (categorical variable, as Table 1), and BMI (categorical variable), self-reported diabetes mellitus, and self-reported hypertension. When one of these possible confounders was the variable of interest, the adjustments were made for the other possible confounders. None of the diabetes and hypertension variables were adjusted for self-reported diabetes and self-reported hypertension, respectively.

c The reference groups included those who did not have the respective condition.

There was an association between BMI and CKD**–**S3-5 in crude analyses. Underweight individuals were at lower risk than participants with normal BMI (OR = 0.21; 95% CI 0.05–0.90), while overweight (OR = 1.86; 95% CI 1.25–2.75) and obese (OR = 2.19; 95% CI 1.54–3.13) participants were at higher risk. After adjustments for age and sex, the association was attenuated but was still significant for obese individuals (OR = 1.78; 95% CI 1.04–3.05); *P* value for trend was 0.01. The association for obese participants disappeared after further adjustment for history of diabetes.

All categories of diabetes that are shown in Table 3 were associated with CKD**–**S3-5 in crude analyses. After adjustment for age and sex, however, only self-reported diabetes showed an association (OR = 1.70; 95% CI 1.00–2.86). After adjustments for BMI and other factors, none of the diabetes categories were associated with CKD**–**S3-5. Similarly, all groups of hypertension were associated with CKD**–**S3-5 in crude analyses. The association for those who had high blood pressure at the baseline examinations disappeared in adjusted models. The association for self-reported hypertension remained after adjustment for age and sex (OR = 3.16; 95% CI 2.02–4.95) or after extension of adjustments for other factors (OR = 2.85; 95% CI 1.77–4.59). Both ischemic heart disease (OR = 2.69; 95% CI 1.14–6.32) and myocardial infarction (OR = 2.73; 95% CI 1.55–4.81) had significant association with CKD in the models adjusted for age and sex. The associations became non-significant after further adjustments for BMI, diabetes, and hypertension.

## Discussion

Our study showed that approximately 5% of inhabitants in Golestan aged 18 years or older had CKD**–**S3-5, with much higher rates among the elderly. We found an association between CKD–S3-5 and BMI and self-reported diabetes and hypertension. Men were at lower risk of CKD–S3-5 than women. Our study also showed high prevalence of diabetes mellitus and hypertension in Golestan.

The prevalence of CKD–S3-5 in our study is similar to the rates reported by NHANES (1988–1994), which was 4.7% [12], and many studies in other populations [20], [21], [22], [23]. A part of the studies, even within a country, have reported very different rates [24]. For example, prevalence of CKD–S3-5 was reported as 18.7% in Japanese general population [25] and as 18.9%, 7.8%, and 6.5% in 3 other studies from Iran [10], [26], [27]. This can be related to true differences in prevalence of the disease in different populations, variation in the methodology that was used, and differences in demographic characteristics of study participants, including age structure. As expected, prevalence of CKD–S3-5 in older ages was higher in this study. This finding is comparable with results of NHANES study, in which prevalence of moderately or severely decreased kidney function in age group over 70 years was 20.6% [12]. Similar to many other developing regions, the age structure pyramid of the population in Golestan is highly broad-based, with a large proportion of young people. Therefore, we expect a rapid increase in the proportion of elderly people and prevalence of CKD in Golestan within a few decades.

BMI is associated with CKD [28]. The observed risk associated with BMI ≥30 in our study is similar with the risk reported in a recent Iranian study (1.8-fold increase in risk) [10]. The association of CKD with BMI may be related to higher prevalence of diabetes among overweight and obese individuals. The association disappeared after adjustment for history of diabetes and hypertension. This suggests that high BMI may be a risk factor for CKD–S3-5 only when it is associated with chronic disorders.

Diabetes and hypertension are known risk factors for CKD [12], [13], [14]. It has been estimated that diabetes and hypertension are responsible for 40.2% and 24.6% of CKD, respectively, in the United States [29]. An earlier study in Iran reported 3.8- and 2.6-fold increased risk of CKD associated with diabetes and hypertension, respectively [10]. The risk associated with self-reported diabetes and hypertension in our study was 1.7-fold and 3.2-fold, respectively. Individuals that were found to have diabetes and hypertension only in baseline examinations were at lower risk compared to those with self-reported disease. It is very likely that the first group had subtle or recently-onset disease. Therefore, their disease might not have enough severity or duration to significantly influence the kidney function. This may explain the difference observed in the association of CKD with self-reported and accidentally-found diabetes and hypertension in our study. As expected, the association of CKD with diabetes and hypertension was attenuated after adjustment for BMI, which is strongly correlated with diabetes and is one of the risk factors of hypertension.

The observed prevalence of diabetes in our population (21.6%) is higher than the world prevalence of diabetes among adults (aged 20–79 years), which is estimated to be 6.4% in 2010 [30]. The reported prevalence rates for diabetes and hypertension in other areas of Iran varies considerably. Among studies on CKD in Iran, the reported prevalence in three studies was 9.2%, 13.7%, and 29.0% for diabetes and 9.9%, 25.8%, and 17.4% for hypertension [10], [26], [27]. However, in a recent national surveillance on approximately 30,000 Iranians aged 15–64 years, prevalence of diabetes and hypertension was reported as 17.4% and 3.5%, respectively [31]. A part of difference in prevalence rates may be related to variations in the demographic structure of studied populations; because, for example, diabetes and hypertension are more common in the elderly and in urban than rural dwellers [32], [33]. In our study, selection bias cannot also be ruled out; individuals with health problems, including diabetes and hypertension, might have been keener to participate in the study. Prevalence of diabetes and hypertension in Golestan may be estimated more accurately in future within the Golestan Cohort Study; a recent large-scale prospective study with approximately 50,000 participants, which was basically designed to study gastrointestinal cancers [34], [35].

In our study, women were at higher risk of CKD than men. Such an association has been reported in several other studies [24], [26], [27], [36]. An earlier study in Golestan reported much higher BMI among women than in men. However, after adjustments for BMI, women were still at higher risk of CKD. There is no clear explanation for this association. However, it may partly be related to residual confounding from BMI and sub-clinical diabetes. Further studies are warranted in this regard.

Fairly large number of participants and collection of data on several factors and medical conditions that enabled us to adjust our results for several potential confounding factors are main strengths of our study. On the other hand, estimating GFR from serum creatinine level has well-recognized limitations, including substantial variation in creatinine production by age, sex, and race [37]. To minimize the impact of these limitations we used MDRD equation, which seems to have higher precision to estimate GFR compared to other methods that are based on serum creatinine [19]. However, serum creatinine values in our study were not calibrated against the values of the laboratory in which data for the development of MDRD equation were generated [38]. Selection bias might have happened in the first call of participants, but not in the second call, which had a high participation rate. The bias might have influenced the reported prevalence rates of disorders, including CKD–S3-5, diabetes, and hypertension. However, it is unlikely that the bias distorted the association between CKD–S3-5 and its risk factors.

In conclusion, aging and high prevalence of obesity, diabetes, and hypertension in Golestan are alarming signs that indicate the need for preventive measures in the region. These may include early diagnosis and treatment of CKD and elimination or control of its risk factors; e.g., programs for weight control and early diagnosis and treatment of diabetes and hypertension. The preventive measures can also be beneficial for several common chronic diseases, including cardiovascular disease. Further studies in the region are required to explain the higher risk observed among women, role of some other risk factors, including insulin resistance and metabolic syndrome [39], and prevalence and risk factors of mild CKD, for which markers of kidney damage, such as albuminuria, should be examined. Screening for CKD in the general population may not be feasible or cost-effective, but it may be considered for those with certain risk factors of CKD [40], including diabetes and hypertension, particularly among the elderly and women. Golestan Cohort Study may provide invaluable material to investigate risk factors of CKD prospectively and to optimal risk prediction of future ERSD in the region.

## Supporting Information

Table S1 Glomerular filtration rate (calculated using Cockroft-Goult method) by demographic characteristics and BMI. Values are numbers (percentages) of participants unless stated otherwise.
(0.04 MB DOC)
