# Supplementary material for: Renal Function and Risk Factors of Moderate to Severe Chronic Kidney Disease in Golestan Province, Northeast of Iran
Source: PLoS One. 2010 Dec 3;5(12):e14216. doi: 10.1371/journal.pone.0014216 (PMC2997063; doi:10.1371/journal.pone.0014216)
Supplement: Table S1 — Glomerular filtration rate (calculated using Cockroft-Goult method) by demographic characteristics and BMI. Values are numbers (percentages) of participants unless stated otherwise. (0.04 MB DOC) [file pone.0014216.s001.doc]

**Supplementary Table 1.** Glomerular filtration rate (calculated using Cockroft-Goult method) by demographic characteristics and BMI. Values are numbers (percentages) of participants unless stated otherwise

|  | **Total no.** | **GFR ≥ 90** | **GFR: 60-89** | **GFR: 30-59** | **GFR: 15-29** | **GFR < 15** |
| --- | --- | --- | --- | --- | --- | --- |
| **All participants** | 3591 | 1983 | 1379 | 220 | 4 | 5 |
| **Women** | 2192 | 1393 | 697 | 100 | 1 | 1 |
| **Men** | 1399 | 590 | 682 | 120 | 3 | 4 |
| **Age (years)** |  |  |  |  |  |  |
| < 30 | 847 | 768 | 74 | 4 | 0 | 1 |
| 30-39 | 656 | 538 | 117 | 1 | 0 | 0 |
| 40-49 | 634 | 373 | 256 | 5 | 0 | 0 |
| 50-59 | 806 | 250 | 531 | 22 | 2 | 1 |
| 60-69 | 433 | 45 | 301 | 85 | 1 | 1 |
| ≥ 70 | 215 | 9 | 100 | 103 | 1 | 2 |

Abbreviations: BMI, body mass index (kg/m^2^); GFR, glomerular filtration rate (ml/mi)
